# Catalytic Activity of Oxidized Carbon Waste Ashes for the Crosslinking of Epoxy Resins

**DOI:** 10.3390/polym11061011

**Published:** 2019-06-07

**Authors:** Enrica Stasi, Antonella Giuri, Maurizio La Villetta, Domenico Cirillo, Gaetano Guerra, Alfonso Maffezzoli, Eleonora Ferraris, Carola Esposito Corcione

**Affiliations:** 1 Dipartimento di Ingegneria dell’Innovazione Università del Salento, 73100 Lecce, Italy; enrica.stasi@unisalento.it (E.S.); antonella.giuri@unisalento.it (A.G.); alfonso.maffezzoli@unisalento.it (A.M.); 2 C.M.D. Costruzioni Motori Diesel S.p.A., Via Pacinotti, 2, 81020 San Nicola La Strada (CE), Italy; maurizio.lavilletta@cmdengine.com (M.L.V.); domenico.cirillo@cmdengine.com (D.C.); 3 Dipartimento di Chimica e Biologia, Università di Salerno, 84084 Fisciano (SA), Italy; gguerra@unisa.it; 4 Department of Mechanical Engineering, Campus de Nayer, 2860 KU Leuven, Belgium; eleonora.ferraris@kuleuven.be

**Keywords:** carbon-based ashes, gel time, differential scanning calorimetry

## Abstract

In this study, two different fillers were prepared from carbon-based ashes, produced from the wooden biomass of a pyro-gasification plant, and starting from lignocellulosic waste. The first type was obtained by dry ball-milling (DBA), while the second one was prepared by oxidation in H_2_O_2_ of the dry ball-milled ashes (oDBA). The characterization of the fillers included wide-angle x-ray diffraction (WAXD), thermogravimetric, and Fourier-transform infrared spectroscopy (FTIR) analysis. The DBA and oDBA fillers were then tested as possible catalysts for the crosslinking reaction of a diglycidyl ether of bisphenol A (DGEBA) with a diamine. The cure reaction was studied by means of rheometry and differential scanning calorimetry (DSC). The oDBA filler exhibits both a higher catalytic activity on the epoxide–amine reaction than the DB*A* sample and improved mechanical properties and glass transition temperature. The results obtained indicate, hence, the potential improvement brought by the addition of carbon-based waste ashes, which allow both increasing the flexural properties and the glass transition temperature of the epoxy resin and reducing the curing time, acting as a catalyst for the crosslinking reaction of the epoxy resin.

## 1. Introduction

Costruzioni Motori Diesel (CMD) developed a wooden biomass pyro-gasification plant, the CMD ECO 20, for the combined production of electrical and thermal energy via thermo-chemical decomposition or molecular dissociation of green wastes at high temperature (from 600 to 1000 °C), in complete absence or minimum quantities of oxygen. This emerging technology improves the efficient use of energy and reduces the environmental impact, by containing the consumption of primary energy and emission of the associated greenhouse gas; in addition, it complies with social aims to decentralize the energy supplied in rural area [1,2]. However, the CMD ECO 20 system produces carbon-based waste, in the form of ashes, the disposal of which constitutes an economic and environmental burden for the company. This paper proposes an original and advantageous re-use of these carbon ashes.

Currently, the potential uses of carbon-based ashes mostly include soil amendment and fertilization methods, followed by the production of construction materials and sorbents, and the synthesis and production of ceramics and other materials [3]. Several studies [4,5,6,7,8] reported the application of the carbon-based ashes, generated mostly via the combustion of biomass, as fertilizer of forest and agricultural soils. However, some ashes should be avoided for soil application because they are commonly highly contaminated with hazardous trace elements such as As, B, Ba, Cd, Cr, Cu, Hg, Mn, Mo, Ni, Pb, Se, Zn, and others. The production of construction materials (especially cement and concrete) currently offers the most valuable opportunities to recycle carbon-based ashes [9,10,11,12]. Carbon-based ashes also possess remarkable adsorption capacity, and some of them, such as those produced via the combustion of wooden biomass, were used and tested as innovative and effective adsorbents for the removal of different elements and compounds from gas emissions and/or wastewater [13,14]. Furthermore, some carbon-based ashes were studied for the potential production of different ceramics [15,16], mineral fibers, and zeolites [15]. 

In this work, an innovative reutilization of carbon-based ashes as catalysts for the crosslinking reaction of epoxy resins with amines is explored. In References [17,18,19,20], the catalytic activities of non-recycled oxidized carbon black (oCB) and graphene oxide (eGO) samples on the kinetics of a reaction of diglycidyl ether of bisphenol A (DGEBA) with a diamine were already deeply analyzed with promising results. Nevertheless, the purpose of this study is to recycle carbon-based waste, produced from wooden biomass pyro-gasification, and to use those fillers as possible catalysts for the crosslinking reaction of the diglycidyl ether of bisphenol A (DGEBA) resin with amines. To the knowledge of the authors, the use of such a filler for the described purpose is not reported in the literature. Given the particular nature and variable composition of this recycled component (i.e., C, K, Ca, O), a number of preparation steps are necessary. The synthesis of catalysts from waste materials became increasingly popular over the past two decades. Recycling waste materials is highly advantageous, since financial and environmental costs are associated with their disposal. Therefore, the conversion of this residue into a valuable catalyst product could significantly reduce these expenses. 

Carbon-based ashes, generated from wooden biomass combustion, are used as catalysts mainly for oxidation applications [21]. The wood ashes catalytically oxidize hydrogen sulfide (H_2_S) and methanethiol (CH_3_SH) at low temperature (23–25 °C). They have a significantly higher surface area compared to coal ashes, resulting in a higher initial H_2_S removal rate under similar conditions [22]. The wood ashes also catalytically oxidize H_2_S using O_2_, and oxidize volatile organic sulfur compounds and propanol in the presence of ozone [23,24]. Additionally, the wood ashes are low-cost catalysts for the oxidation of 2-methylbutanal and hexane vapors at low temperature (15–160 °C) using oxygen and ozone as oxidants [25]. 

A sulfonated green carbon catalyst was also produced from in situ carbonization and sulfonation of lignocellulosic biomass for the tertiary butylation of phenol, exhibiting comparable catalytic performance up to five reaction cycles [26]. A by-product of the fast pyrolysis of the woody biomass, i.e., biochar, was investigated as a solid acid catalyst for the simultaneous transesterification and esterification of a mixture of canola oil and oleic acid for the production of biodiesel [27]. 

In this study, two fillers were prepared from the carbon-based waste ashes of the CMD ECO 20 pyro-gasification plant. The carbon ashes were firstly ball-milled in dry conditions and then oxidized with a simple and eco-friendly procedure, i.e., by using H_2_O_2_. The oxidized and un-oxidized wastes samples were then characterized via wide-angle x-ray diffraction (WAXD), Fourier-transform infrared (FTIR) spectroscopy, and thermogravimetric analysis (TGA), before being tested as possible catalysts for the crosslinking reaction of a diclycidyl ether of bisphenol A (DGEBA) with a diamine. The neat epoxy and carbon-based ash epoxy, filled with oxidized and un-oxidized fillers, were cured, and the flexural properties of all the samples were measured and compared each other.

Since the possibility to both reinforce and catalyze an epoxy resin by means of carbon-based waste ashes was not yet fully investigated, the biomass waste recycling for this application assumed a primary importance, allowing the reinforcement of the matrix, the decrease of the curing time of the epoxy resin, and the increase of the efficiency of waste management with the reduction of its negative effects on the environment and on the population.

## 2. Materials and Methods

### 2.1. Resin and Curing Agent

The epoxy diglycidyl ether of bisphenol A (DGEBA) resin was purchased from Elantas Electrical Insulation (Collecchio, Italy), with the trade name EC01.

The curing agent isophorondiamine (IPDA) was supplied by Sigma Aldrich (Milan, Italy). According to the stoichiometric ratio, 22 parts of hardener (i.e., 22 phr) was added to 100 parts of the epoxy resin.

### 2.2. Ashes

The ashes were produced by the biomass pyro-gasification plant CMD ECO 20. The company Costruzioni Motori Diesel (CMD, San Nicola la Strada, Italy) developed the CMD ECO 20 system for producing heat and electric power, starting from innovative lignocellulosic waste [1]. 

Two different types of fillers were produced from ashes: the first type was obtained by dry ball-milling of the ashes (DBA), while the second one included water-induced oxidation of the milled particles (oDBA), according to the method described in Reference [28]. Briefly, for the preparation of the DBA, 100 g of ash was milled for 24 h in an aluminous porcelain jar (1.5 L), using alumina balls in ambient atmosphere. The mechanical milling was performed in a horizontal oscillatory mill (MMS-Ball Mill) operating at ±25 Hz. The oxidation of the oDBA fillers was obtained by introducing 500 mg of DBA and 250 mL of H_2_O_2_ in a 500-mL flask; the flask was placed in a thermostat bath at 60 °C, and the solution was kept under magnetic stirring for 24 h. Afterward, about 500 mL of water was added, and the whole mixture was washed by centrifugation for 30 min at 4000 rpm. The fillers were then dried overnight on a hot plate at 60 °C. Table 1 reports the names and preparation procedures of the fillers.

### 2.3. Preparation of Epoxy/Ash Composites

The filled epoxy mixtures were stirred for 5 h at 80 °C and 400 rpm and subsequently degassed under vacuum at T = 60 °C. Then, 22 phr of IPDA was successively added to the filled resin mixtures. A filler amount of oxidized or un-oxidized compounds equal to 3 wt.% was added to the epoxy matrix. Table 2 lists the names and compositions of the mixtures.

### 2.4. Characterization Techniques

#### 2.4.1. Wide-Angle X-Ray Diffraction

Wide-angle X-ray diffraction (WAXD) patterns of the fillers before and after oxidation processing were obtained using an automatic Bruker D2 Phaser diffractometer (Billerica, MA, USA), in reflection mode, at 35 KV and 40 mA, using nickel-filtered Cu-Kα radiation (1.5418 Å).

#### 2.4.2. Infrared Spectroscopy

FTIR spectra of the fillers before and after oxidation processing were obtained with a BRUKER Vertex70 spectrometer (Billerica, MA, USA) equipped with a deuterated triglycine sulfate (DTGS) detector and a KBr beam splitter, at a resolution of 2.0 cm^−1^. The frequency scale was calibrated to 0.01 cm^−1^ using an He–Ne laser. In total, 32 scans were signal averaged to reduce the noise. Spectra of powder samples were collected using KBr pellets.

#### 2.4.3. Thermogravimetric Analysis

Thermogravimetric analysis (TGA) of the fillers before and after oxidation processing was performed using a TGA TA instrument SDT Q600 (TA Instrument, New Castle, DE, USA). About 10 mg of powder samples was heated in an alumina holder under nitrogen atmosphere from 20 to 600 °C at a heating rate of 10 °C/min.

#### 2.4.4. Rheometry

The rheological characterization of the formulations was carried out in a strain-controlled rheometer (Ares TA Instrument, New Castle, DE, USA), adopting a parallel plate geometry with plates of 12.5 mm in radius. The evolution of the storage modulus, G′, and loss modulus, G″, of the unfilled and filled epoxy mixtures was evaluated as a function of time. Three isothermal tests at 50 °C were performed on each sample using a frequency of 1 Hz and a deformation of 10%. The dynamic–mechanical properties of a curing system depend on the degree of reaction, and dramatically change when the gel point approaches. The time to gelation (or gel time, t_gel_) was determined according to the literature, and specifically as the cross-point between the G′ and G″ curves [20,29,30]. Each test was performed until gelation was observed. It must be underlined that the gel time is observed for a constant value of the degree of reaction, according to Flory [31]; hence, it represents a fast way to compare the rate of reaction of thermosetting resins with the same reagents and under different conditions, such as temperature or catalyst.

#### 2.4.5. Differential Scanning Calorimetry

The curing reaction of the filled and unfilled epoxy resins was measured using a differential scanning calorimeter (DSC) supplied by Mettler Toledo 622 (Columbus, OH, USA). Isothermal DSC scans were performed on liquid epoxy mixtures at 50 °C for 5 h and under nitrogen atmosphere. Three repetitions were performed for each sample. The reaction was considered to be complete when the heat flow curve reached a constant value. The area under the exothermal curve was used to calculate the heat of reaction, ∆H (J/g), at the test temperature. The calculation was based on an extrapolated horizontal baseline aligned to the asymptotic value of the DSC signal at the end of the reaction. Dynamic DSC scans were also performed from 20 °C to 250 °C every 10 °C /min, under nitrogen atmosphere [20]. 

The rate of curing reaction, dα/dt, was calculated from the DSC scan as

(1) $$\frac{d\alpha }{dt}=\;\frac{1}{\Delta H_{U}}\left(\frac{dH}{dt}-BL\right),$$

where BL is the baseline, set as described above, dH/dt is the heat flow for unit mass, ∆H_U_ is the total heat of the reaction obtained by the integration of each peak of the dynamic scan, adopting the same baseline [20,32], and α represents the degree of reaction.

Cole and Kenny et al. [20,33,34] proposed a simple method to explain vitrification as the cause of the end of a reaction at low curing temperature, according to Equation (2) [20,35,36].

(2) $$\frac{d\alpha }{dt}=(k_{1}+k_{2}\alpha^{m}){\left(\alpha_{max}-\alpha \right)}^{n},$$

where α_max_ is the maximum degree of cure at a given temperature due to a vitrification observed during isothermal cure. The constants m and n are the reaction powers, which are independent of temperature, and they are experimentally determined; k_1_ and k_2_ are temperature-dependent rate constants. The isothermal rates of the reaction data were interpolated with Equation (2), calculating α_max_ from

(3) $$\alpha_{max}=\frac{\Delta H_{max}}{\Delta H_{u}},$$

where ∆H_max_ is the maximum heat of the curing reaction calculated from the isothermal scan, and ∆H_u _is the heat released during complete curing, measured from the dynamic scan.

#### 2.4.6. Flexural Tests and Glass Transition Temperature measurements

The mixtures listed in Table 2 were cured for 1 h at 60 °C and 2 h at 150 °C, according to the curing cycle assessed in previous papers [37,38]. The flexural properties of each cured sample were measured using a dynamometer, Lloyd LR5K, according to the ASTM D790 (three-point bending with specimen dimension 80 × 10 × 4 mm). Five repetitions were performed on each sample. 

The glass transition temperature of the cured neat epoxy and composites was measured using a differential scanning calorimeter (DSC; Mettler Toledo 622, Columbus, OH, USA). The cured samples were heated from 20 to 250 °C at 10 °C/min under nitrogen atmosphere, and at least three tests on each sample were performed.

## 3. Data Processing and Experimental Results

### 3.1. Characterization of Ash Fillers

#### 3.1.1. Wide-Angle X-Ray Diffraction

**Figure 1:**
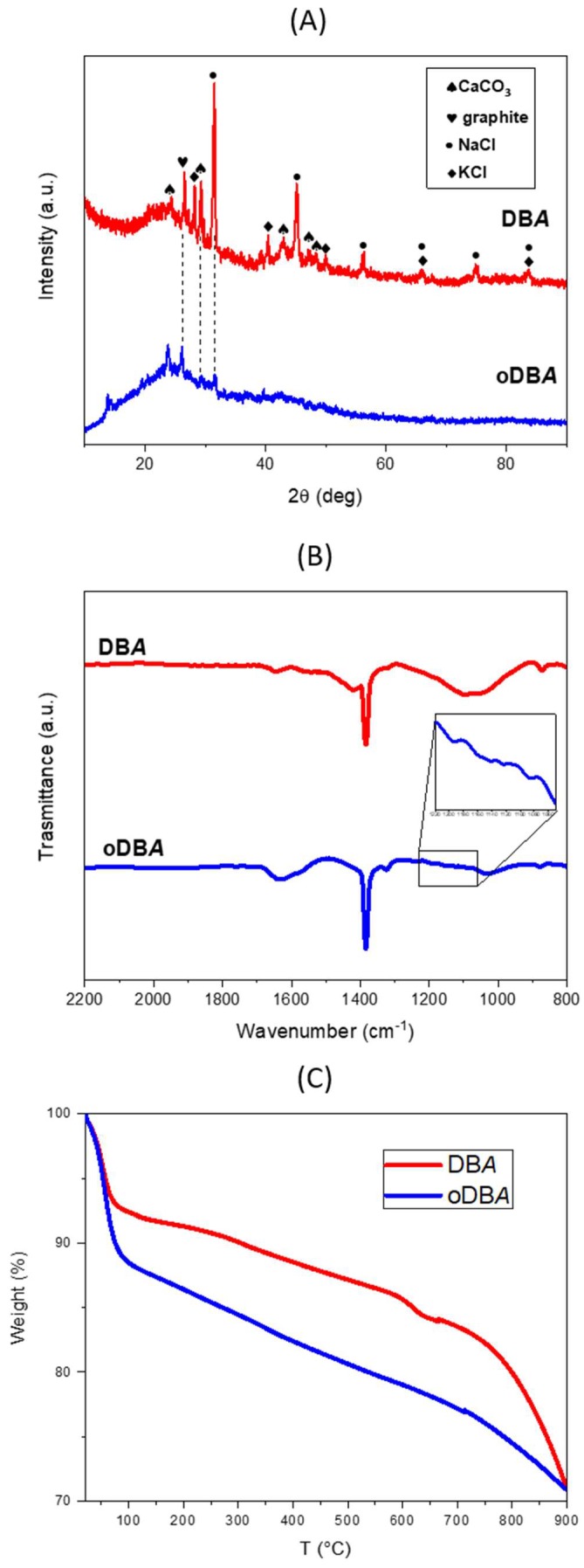
(**A**) X-ray diffraction of dry ball-milled ashes (DBA; blue curve) and oxidized dry ball-milled ashes (oDBA; red curve); (**B**) Fourier-transform infrared (FTIR) spectra of DBA before and after H_2_O_2_ treatment; (**C**) thermogravimetric analysis (TGA) scans of un-oxidized and oxidized DBA.

The structure of the ashes was obtained by WAXD as shown in Figure 1A, which compares the WAXD patterns of DBA and oDBA. The DBA curve shows several crystalline peaks which can be attributed to potassium chloride (KCl), sodium chloride (NaCl), and calcium carbonate (CaCO_3_), and only one peak at 2θ = 26.5°, which is related to graphite. In the oDBA curve, the graphitic peak at 2θ = 26.5° remains, while the other peaks are very small or missing.

#### 3.1.2. Infrared Spectroscopy

The chemical nature of the oxidized groups was studied by Fourier-transform infrared (FTIR) spectroscopy. The FTIR spectra of the DBA before and after treatment with H_2_O_2_ at 60 °C are shown in Figure 1B. In particular, in the oDBA spectrum, many weak vibrational peaks appear in the 1220–1050-cm^−1^ region which are associated with the stretching of C–O single bonds, thus confirming the oxidation of the ashes. 

#### 3.1.3. Thermogravimetric Analysis

A qualitative confirmation of the oxidation of DBA ashes was determined by TGA analysis, as reported in Figure 1C. 

The weight loss profiles in the TGA curves entailed two main steps for both samples. The first step, ranging from room temperature to about 100 °C, is attributable to the removal of the molecularly adsorbed water. DBA exhibited a weight loss of about 8 wt.%, while the oxidized DBA underwent a slightly higher loss of about 12 wt.%. This indicates that the presence of oxygen-containing groups in the oxidized filler increases the ability of the DBA surface to absorb water. Indeed, in the second step, developing between 100 and 250 °C, originating from the removal of the thermally labile oxygen-containing functional groups, the DBA exhibited a weight loss of about 2 wt.%, achieving a plateau around 250 °C, compared to the 4% observed for the oDBA, which showed a continuous decrease in weight. The analysis confirmed the presence of a certain number of oxygen groups in the oxidized ashes. 

### 3.2. Characterization of the Liquid Ash–Epoxy Mixtures

**Figure 2:**
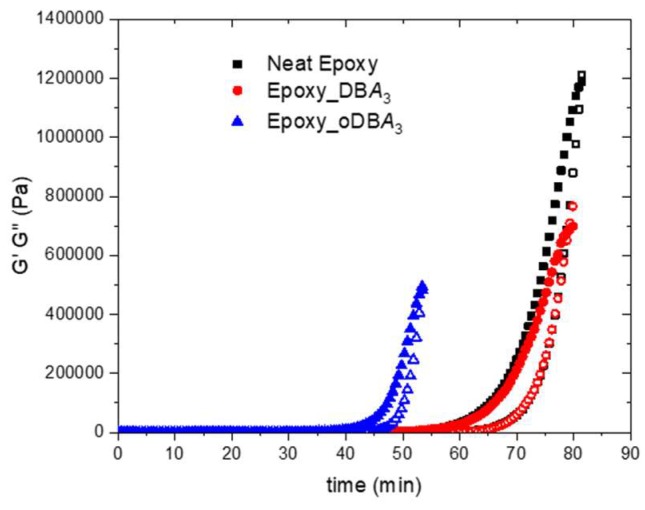
Storage (G′, hollow symbols) and loss modulus (G″, solid symbols) versus time for the pure EC01–isophorondiamine (IPDA) epoxy resin, (black color) and the 3 wt.% filler-based solutions: DBA and oDBA (red and blue color, respectively).

The ash fillers characterized in the previous section were tested as possible catalysts for the crosslinking of the epoxy resin EC01 with amines. The results of rheological and calorimetric measurements of the epoxy–IPDA mixtures containing 3 wt.% of each filler are here reported. The cure reaction was studied by isothermal rheological measurements at 50 °C. The storage modulus, G′ (hollow symbols), and the loss modulus, G″(solid symbols), of unfilled and filled compounds, as determined by forced harmonic oscillation measurements, are reported in the Figure 2 as a function of time.

Table 3 reports the gel time (**t_gel_**) of each mixture. It is identified as the cross-point of the G′ and G″ curves of the neat resin and composite mixtures.

The gel time of the neat resin (**t_gel_**= 81.3 min) remained approximatively constant when DBA was added (**t_gel_** = 79.0 min), while it significantly reduced in the presence of oDBA (**t_gel_** = 53.3 min). These data suggest a catalytic activity of the oxidized filler on the epoxy crosslinking.

**Figure 3:**
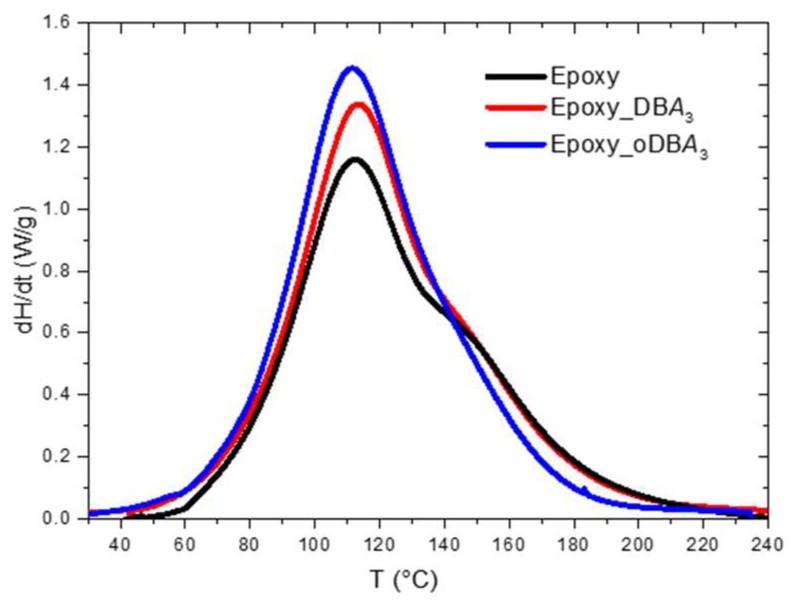
Differential scanning calorimetry (DSC) spectra of the neat epoxy resin and composite resins with 3 wt.% DBA and oDBA.

Figure 3 reports the DSC spectra of the neat epoxy composite resins (3 wt.% filler solutions) at a heating rate of 10 °C/min.

Next to the principal exothermic peak at 113 °C, the spectrum of the neat resin (black curve in Figure 3) presents a shoulder located at about 145 °C; it could be due to a hydroxyl–epoxy addition (etherification) reaction [18]. Similarly, the DBA 3 wt.% composite displays a higher exothermic peak located at 114 °C and a shoulder at 145 °C. The intensity of the exothermic peak at 112 °C increased with the presence of the oDBA filler. The DSC dynamic results are summarized in Table 4.

**Figure 4:**
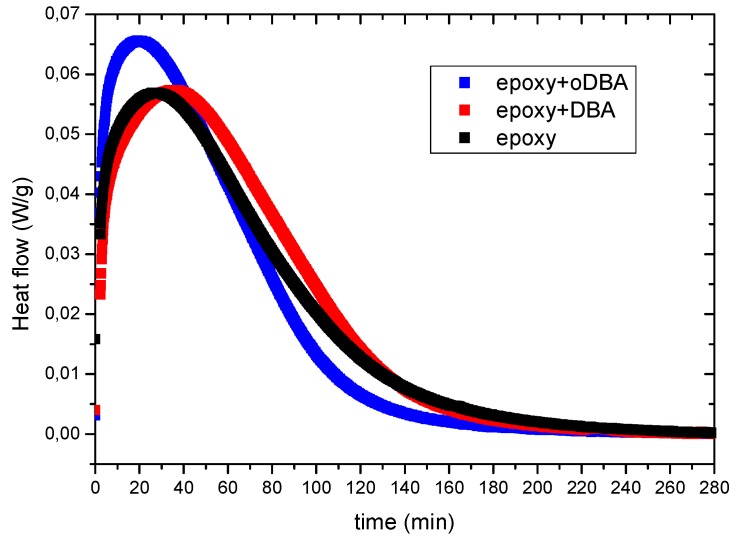
Isothermal DSC scans at 50 °C of the EC01–IPDA epoxy resin. The lowest curve (black) corresponds to the neat epoxy resin. The other curves correspond to the epoxy resin filled with 3 wt.% DBA (red) and oDBA (blue curve).

Figure 4 reports the isothermal DSC scans of the neat epoxy and composite resins at 50 °C. A short time interval, usually about 130 s, is needed for the temperature stabilization after the ramp at 40 °C/min used to reach the isothermal test temperature. The transition between the dynamic and isothermal conditions is responsible for a jump in the heat flow, which makes the DSC data obtained in the first 130 s not reliable. For this reason, all isothermal curves were corrected, neglecting the data points in the first 130 s and determining by linear extrapolation of the first three remaining data points the value of the heat flow at time = 0. These corrected isothermal DSC scans are shown in Figure 4, and the isothermal heat of reaction and the time to reach the peak are calculated according to the procedure described in Section 2.4.5, and are listed in Table 5 along with the glass transition temperature (**T_g_**), measured from a dynamic scan performed after the isothermal scan [20].

As already observed for the gel time data of Figure 2, the peak time (**t_peak_**) of Figure 4 strongly decreased in presence of the oDBA filler. This confirms the gel time data, indicating that this filler has a catalytic activity on the epoxide–amine reaction. The catalytic effect of oDBA is more evident in the isothermal DSC experiment at 50 °C rather than in the dynamic scan at 10 °C/min. This could be explained accounting for a reduction of the oDBA upon heating above 50 °C, which reduces its efficiency as catalyst [28]. According to Figure 3, most of the reaction heat in the dynamic DSC scan is developed when the sample is above 90 °C, probably when the chemical composition of ashes is changed. However, the experimental data reported in Table 5 highlight that the catalyst has no relevant effect on both characteristics: the total heat of reaction of the epoxy resin and the glass transition temperature (T_g_) measured after the isothermal scan at 50 °C. This latter result indicates that, despite its catalytic activity, the carbon-based filler does not remarkably affect the maximum degree of reaction, but only the curing time. The maximum degree of reaction at 50 °C is due to vitrification, which determines the end of the reaction through a dramatic decrease of the molecular mobility, a diffusive effect, not related to the consumption of the reactive groups. However, a slight increase in crosslinking density is obtained when DBA is added, as indicated by a slight increase of **T_g_**.

**Figure 5:**
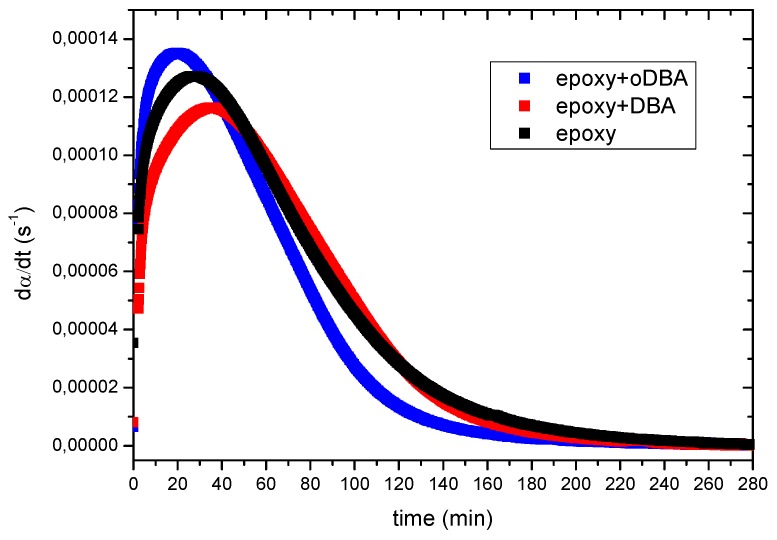
The dα/dt curves calculated from the DSC data shown in Figure 4 using Equation (1) (Section 2.4.5).

Figure 5 shows the dα/dt experimental curves obtained by applying Equation (1) to the DSC isothermal scans of each system.

**Figure 6:**
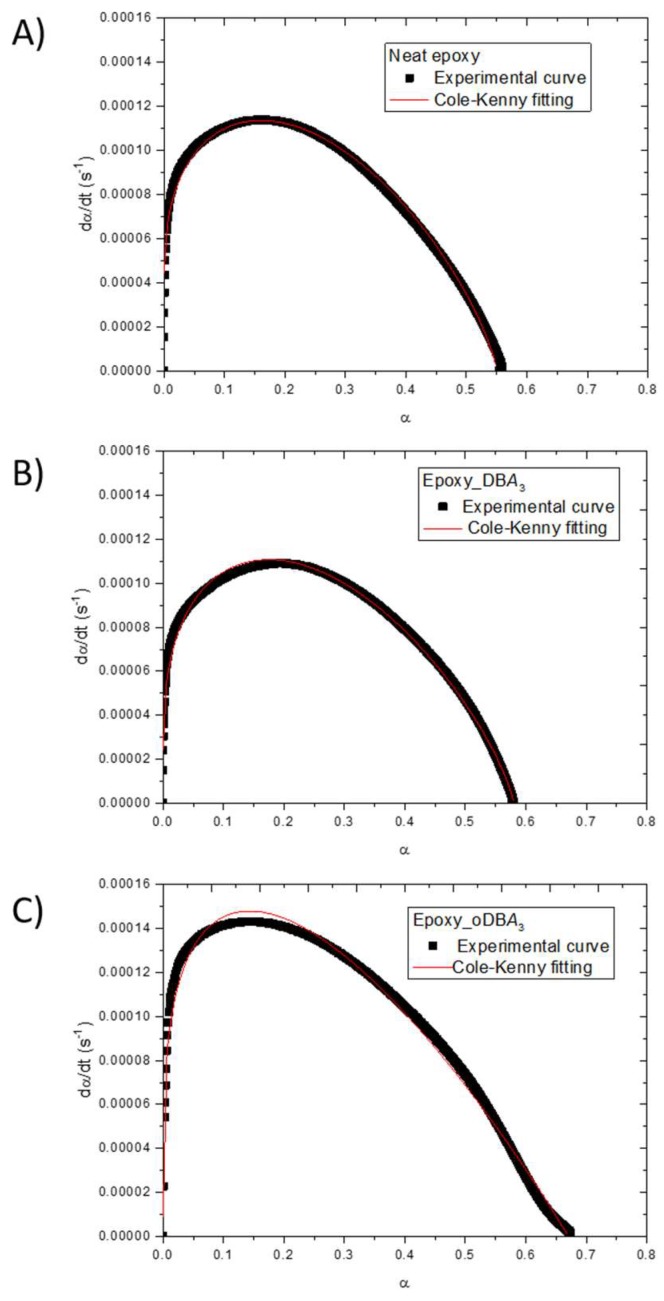
Comparison between the experimental curves and the Equation (2) model predictions of (**A**) neat epoxy resin and epoxy resin filled with 3 wt.% (**B**) DBA and (**C**) oDBA.

On the contrary, Figure 6 reports a comparison between the experimental curves and the kinetic model predictions.

The constant k_1_ constitutes the initial rate of the reaction. Table 6 compares the dα/dt peaks, and model parameters of the neat epoxy and composite resins with those of epoxy resins containing 3 wt.% oxidized carbon black with a specific surface area of 36 (oCB-1), 125 (oCB-2), and 151 m^2^/g (oCB-3), and exfoliated graphite oxide (eGO), as previously reported [20]. 

The parameter m in Equation (2) is associated with the increase of the reaction rate at the beginning of the reaction (low values of α) and before the peak, while the parameter n in Equation (2) prevails in the final part of the reaction (high values of α) [39]. The lower value of m, observed when oDBA is used, indicates that the autocatalytic effect is higher than that of the neat epoxy. This value is comparable to that observed in the presence of the different oxidized carbon blacks (oCB-1, oCB-2, oCB-3) and graphene oxide (eGO) with the same resin composition [20], as also reported in Table 6. 

### 3.3. Characterization of the Cured Samples

**Figure 7:**
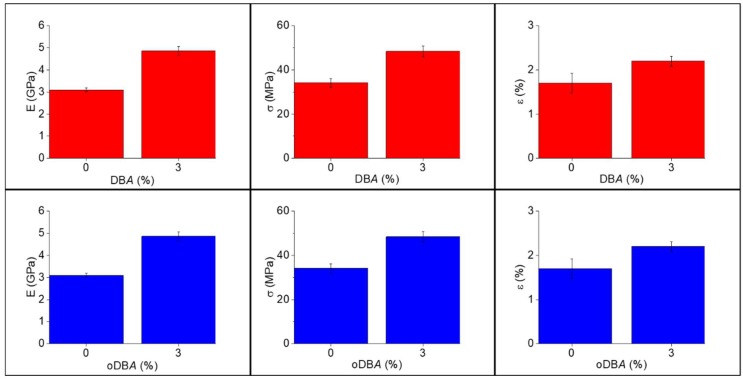
Flexural modulus (E), strength (σ), and strain to break (ε) of the epoxy composites as a function of the content of DBA and oDBA and compared to neat epoxy samples.

Table 7 lists the flexural modulus (E), strength (σ), strain to break (ε), and glass transition temperature (T_g_) of the neat epoxy and epoxy resins containing 3 wt.% DBA or oDBA, obtained according to the procedure described in Section 2.4.6. The Figure 7 shows that the flexural modulus increases by about 50% when 3 wt.% DBA or oDBA filler is added as compared to the neat epoxy samples. Similarly, the strength and strain to break improve in the presence of the fillers. The oxidized filler does not increase the maximum degree of reaction of the epoxy resin, but only decreases the curing time. Hence, when adding oDBA*,* the flexural properties do not improve compared to those in the presence of DBA. Furthermore, the T_g _of the epoxy matrix increases from 148.0 °C to 156.3 °C when the fillers are added, irrespective of the oxidation of the fillers, confirming that they have either (i) a reinforcing effect on the epoxy matrix or (ii) the capability to promote a higher T_g_, probably as a consequence of the induced faster kinetics.

## 4. Conclusions

The effect of the un-oxidized (DBA) and oxidized (oDBA) carbon waste ashes as possible catalysts of the crosslinking reaction of the diglycidyl ether of bisphenol A (DGEBA) resin with amines was studied. 

The oxidation of DBA was obtained with a simple and eco-friendly procedure using H_2_O_2_, and the FTIR and TGA results confirmed the successful DBA oxidation reaction.

The rheological data at 50 °C attest to the occurrence of a catalytic effect of the oxidized waste sample toward the epoxy curing reaction. In fact, the gel time of the neat epoxy resin was reduced by 52% in the presence of 3 wt.% oDBA. 

Similarly, the isothermal DSC studies at 50 °C show that the peak time is reduced by 40% when oDBA is added. The application of an autocatalytic model to the experimental DSC data of the unfilled and filled epoxy resin led to kinetics parameters which are coherent with the predicted catalyzed reaction.

The obtained results confirm that the oxidized ash filler in the epoxy composites acts as a catalyst, reducing the crosslinking (curing) time of the thermoset matrix. These results are comparable with those reported in Reference [20] relative to the catalytic activity of oxidized carbon black (oCB) and graphene oxide (eGO) for the crosslinking of epoxy resin. In detail, the gel time of the composite epoxy resin decreases in the presence of the same amount of the oDBA filler. Furthermore, the curing time of the epoxy matrix was strongly reduced when 3 wt.% oDBA was added, and this result is similar to that obtained for the epoxy resin containing the same amount of oCB (125 m^2^/g) and eGO [20]. On the other hand, the carbon-based waste filler used in this paper, unlike the other carbon-based fillers previously used, showed a doubly important role, i.e., reinforcing and catalyzing the crosslinking reaction of the diglycidyl ether of bisphenol A (DGEBA) resin with amines, evidencing, in turn, a further advantage in addition that of being a low-cost recycled material, in comparison to other kinds of carbon-based fillers.

In conclusion, the experimental results obtained in this paper show the potential of the developed approach for the recycling of waste carbon-based ashes, by producing epoxy composite samples characterized by higher mechanical properties compared to epoxy resin, as well as a cost reduction and an increase in the efficiency of waste management, with the reduction of its negative effects on the environment and on the population.

## Figures and Tables

**Table 1:** Names and preparation procedures of the carbon-based fillers.

Name	Preparation procedure
DB*A*	Dry ball-milled ashes
oDB*A*	Oxidized dry ball-milled ashes

**Table 2:** Names and compositions of mixtures. IPDA—isophorondiamine.

Name	Weight Composition
Neat epoxy	82% epoxy resin + 18% IPDA
Epoxy_DB*A*_3_	79.6% epoxy resin + 3% of DB*A*+ 17.4% IPDA
Epoxy_oDB*A*_3_	79.6% epoxy resin + 3% of oDB*A*+ 17.4% IPDA

**Table 3:** Gel time (**t_gel_**) measured as the cross-point of the G′ and G″ curves of the neat resin and composite mixtures.

Name	t_gel_
Neat epoxy	81.3 ± 1.1
Epoxy_DBA_3_	79.0 ± 0.92
Epoxy_oDBA_3_	53.3 ± 0.86

**Table 4:** Results of differential scanning calorimetry (DSC) scans at 10 °C/min, for the neat epoxy resin and epoxy resin with 3 wt.% DBA and oDBA: enthalpy changes, normalized with respect to the resin weight (**∆H_U_**) and peak temperature (**T_peak_**).

Name	∆H_U_ (J/g)	T_peak_ °C
Neat epoxy	447.2 ± 12.1	112.7
Epoxy_DB*A*_3_	493.0 ± 23.6	113.6
Epoxy_oDB*A*_3_	485.1 ± 21.3	111.5

**Table 5:** Enthalpy, normalized with respect to the resin weight (**∆H**) and peak time (**t_peak_**) obtained by isothermal DSC scans at 50 °C, for the neat epoxy resin and the epoxy resin with DBA or oDBA.

Name	∆H (J/g)	t_peak_ (min)	T_g _(°C)
Neat epoxy	308.04 ± 10.1	27.08 ± 0.2	61.6 ± 3.2
Epoxy_DB*A*_3_	321.50 ± 1.2	35.75 ± 0.5	68.6 ± 0.5
Epoxy_oDB*A*_3_	290.40 ± 22.3	19.28 ± 0.1	64.0 ± 0.7

**Table 6:** The dα/dt_peak _and kinetic model parameters, according to Equation (2), compared to that obtained in the presence of different oxidized carbon blacks (oCB-1, oCB-2, oCB-3) and graphene oxide (eGO) with the same resin composition [20] .

Name	dα/dt_peak_ (s^−1^)	m	n	K_1_ × 10^6^ (s^−1^)	K_2_ × 10^6 ^(s^−1^)	Reference
Neat epoxy	1.27 × 10^−4^	0.35	0.95	35.7	386	This work
Epoxy_DB*A*_3_	1.16 × 10^−4^	0.32	0.81	8.06	367	This work
Epoxy_oDB*A*_3_	1.35 × 10^−4^	0.27	0.91	6.53	470	This work
Epoxy + oCB-1	2.33 × 10^−4^	0.21	1.07	740	340	[20]
Epoxy + oCB-2	3.02 × 10^−4^	0.39	1.22	230	630	[20]
Epoxy + oCB-3	4.49 × 10^−4^	0.30	1.58	230	670	[20]
Epoxy + eGO	3.18 × 10^−4^	0.20	1.22	990	490	[20]

**Table 7:** Mechanical properties and glass transition temperature of the epoxy composites obtained after dynamic DSC scans up to 250 °C.

Name	E (GPa)	σ (MPa)	ε (%)	T_g_ (°C)
Neat epoxy	3.10 ± 0.09	34.10 ± 2.0	1.7 ± 0.22	148.0 ± 2.0
Epoxy_DB*A*_3_	4.86 ± 0.24	48.42 ± 2.4	2.2 ± 0.11	156.3 ± 1.3
Epoxy_oDB*A*_3_	4.86 ± 0.24	48.42 ± 2.4	2.2 ± 0.11	156.3 ± 1.6
